# Antique Traditional Practice: Phenolic Profile of Virgin Olive Oil Obtained from Fruits Stored in Seawater

**DOI:** 10.3390/foods9101347

**Published:** 2020-09-23

**Authors:** Jelena Torić, Monika Barbarić, Stanko Uršić, Cvijeta Jakobušić Brala, Ana Karković Marković, Maja Zebić Avdičević, Đani Benčić

**Affiliations:** 1 Faculty of Pharmacy and Biochemistry, University of Zagreb, A. Kovačića 1, 10000 Zagreb, Croatia; jelenatoric@gmail.com (J.T.); dr.sursic@gmail.com (S.U.); cjakobus@pharma.hr (C.J.B.); akarkovic@pharma.hr (A.K.M.); 2 Faculty of Mechanical Engineering and Naval Architecture, University of Zagreb, Ivana Lučića 5, 10000 Zagreb, Croatia; maja.zebic@fsb.hr; 3 Faculty of Agriculture, University of Zagreb, Svetošimunska cesta 25, 10000 Zagreb, Croatia; bencic@agr.hr

**Keywords:** HPLC analysis, olive oil, phenolic compounds, seawater

## Abstract

Virgin olive oil (VOO) is a functional food specific to the Mediterranean diet and related to human health, especially as a protector of cardiovascular health, in the prevention of several types of cancers, and in modification of immune and inflammatory response. Phenolic compounds have central importance for these extraordinary health benefits. In the production of high-quality olive oils, it is very important to process freshly picked olives and avoid any storage of fruits. However, in Croatia there is a very traditional and environmentally friendly method of olive oil production, where olive fruits are stored in seawater for some time prior to processing. This practice is also notable nowadays since there are people who prefer the characteristic flavor of the “seawater olive oil”, although some people argue against its quality and biomedical relevance. In this study, the phenolic contents of VOO prepared from the immediately processed fresh olives and olives processed after storage in seawater were compared with the use of high-performance liquid chromatography-mass spectrometry (HPLC-MS) and spectrophotometric analysis. The results suggest that “seawater olive oil” should be considered as a safe food of biomedical relevance, as it still contains a significant proportion of important phenolics like hydroxytyrosol, tyrosol and oleacein (e.g., 63.2% of total phenols in comparison to VOO).

## 1. Introduction

Most of the extra virgin olive oils (EVOOs) of the highest quality from the Croatian coastal region and isles of the Adriatic Sea are obtained by pressing freshly picked olives and avoiding any storage of fruits before common mechanical processing. However, although it is well known that immediate processing of olive fruits is an important factor to obtain high quality olive oil, in many cases limited capacities of olive oil mills do not make possible the quick processing of collected olives. A very traditional method of olive oil production in Croatia (the storage of olive fruits in seawater for some time prior to processing into oil) is frequently used in the coastal area and isles. The tradition has persisted in the Adriatic since the Roman Empire [1]. There are people who prefer the characteristic flavor of the “seawater olive oil” (that is, the oil obtained from olives stored for some time in seawater) in contrast to others who have claimed that this olive oil is inferior in any way in comparison with oils obtained using a common modern practice.

Notwithstanding, due to differences in flavor it is customary to have both types of the olive oils at the table frequently, and additionally owing to recently awakened interests of visitors and tourists in the traditionally obtained olive oil, there is an increased demand in the local market for the oil from the seawater stored olives.

Virgin olive oils (VOOs), as is very well known, are not just nutritious but also have health benefits. Many studies suggesting numerous beneficial health effects associated with dietary consumption of EVOOs have steadily accumulated for decades. In this context, to mention only a few issues, the phenolic compounds naturally present in EVOOs are deemed to be of central importance for the beneficial cardiovascular, antiatherogenic/anti-inflammatory, anticancer, neuroprotective, antioxidant and antimicrobial effects [2,3,4,5,6,7,8].

Therefore, when dealing with the differences between the oils from seawater stored olives and the oils obtained using a common modern practice, there is an important question as to whether the phenolic compounds naturally present in olive fruits (and consequently in olive oil obtained by common practice) have been preserved or lost in the “seawater oils”, at least to some extent during the storage in seawater. Intuitively, one would expect that the phenolics contained in olive fruits could be lost extensively due to enzymatic processes expected to take place during the seawater storage. However, to the best of our knowledge there is no study dealing with the question to date. Therefore, to obtain quantitative data needed to reach an informed and reliable insight into the issue, we performed a side-by-side analysis of the phenolic content in the olive oil prepared from fresh olive fruits immediately after harvest (VOO) and the “seawater olive oil” (VOO-Sea). The oils were obtained using samples of olive fruits from a batch of the same olive trees of the variety Oblica harvested on the Ugljan isle (central part of Croatian Adriatic, nearby the city of Zadar) in the 2016 season. Oblica is the main variety in the Dalmatian region spread throughout the Mediterranean area of Croatia where it occupies about 60% of all productional olive trees [9]. It should be noted that the Isle of Ugljan was an important point in the olive oil production during the Roman Empire age, continuing up to the olive cultivation and oil production to date.

## 2. Materials and Methods

### 2.1. Reagents and Standards

Methanol (high-performance liquid chromatography, HPLC grade), 2,2-diphenyl-1-picrylhydrazyl (DPPH), catechin, benzoic acid, hydroxytyrosol, oleuropein, homovanillyl alcohol, *p*-coumaric acid, pinoresinol and apigenin were purchased from Sigma-Aldrich Chemie GmbH (Steinheim, Germany). Tyrosol, 3,4-dihydroxybenzoic acid, gallic acid, *p*-hydroxybenzoic acid, syringic acid, vanillic acid, cinnamic acid, ferulic acid, *o*-coumaric acid and Folin-Ciocalteu’s reagent were obtained from Fluka Chemie GmbH (Buchs, Switzerland). Formic acid (99+%) and vanillin were purchased from Acros Organics (Morris Plains, NJ, USA). Sodium hydroxide and sodium carbonate anhydrous were purchased from Kemika (Zagreb, Croatia) while sodium molibdate dehydrate, sodium nitrite, glacial acetic acid and aluminum chloride were obtained from Merck (Darmstadt, Germany).

### 2.2. Olive Oil Samples Preparation

The olive oil samples were obtained using olive fruits from a batch of the same olive trees of the variety Oblica. All olive trees were grown under the same agronomic and environmental conditions on the Ugljan isle (central part of Croatian Adriatic, nearby the city of Zadar). After harvesting in the 2016 season, fresh olive fruits (50.0 kg) were immediately processed by centrifugal line (Olimio, 150 kg, Enologia Mori, Tuscany, Italy) giving virgin olive oil (VOO). The other fresh olive fruits (50.0 kg) were kept in the seawater for ten days and then removed from the seawater and processed on the same line giving “seawater olive oil” (VOO-Sea). All samples were transferred in dark glass bottles without headspace and stored in the dark at 4 °C until analysis.

### 2.3. Phenolic Compounds

#### 2.3.1. Extraction

Extractions of phenolic compounds from VOO and VOO-Sea were carried out using the ultrasound-assisted liquid-liquid extraction (US-LLE) method previously described by Jerman Klen and Mozetič Vodopivec [10] with some modifications. VOO and VOO-Sea (20 g) were dissolved in *n*-hexane (10 mL) and sonicated (3 × 10 min at 25 °C) with methanol (15 mL) using an ultrasound bath (Elma Transsonic T570 HF = 320 W, Singen, Germany). The homogenates of each extraction step were centrifuged (15 min at 4000 rpm) using Hettich zentrifugen D-78532 (Tuttlingen, Germany), combined and defatted by shaking the suspension with hexane. The methanolic extracts were concentrated with rotary evaporator (Büchi Heating Bath B-490, Büchi Labortechnik AG, Flawil, Switzerland) at 38 °C. After successive methanol evaporation, the residue was redissolved in methanol (1.7 mL) and filtered through 0.20 μm/13 mm polytetrafluoroethylen (PTFE) filters (Macherey-Nagel GmbH & Co. KG, Düren, Germany) prior to analyses. Each sample of oil was extracted three times.

#### 2.3.2. HPLC-DAD Analysis

A HPLC-DAD analysis was performed essentially by applying the procedure [11]. The HPLC system (Perkin Elmer Series 200, Shelton, CT, USA) equipped with diode array detector (DAD) was used. Separation was achieved at 25 °C on a C18 Restek (Bellefonte, PA, USA) column (5 μm, 250 × 4 mm). The flow rate was 1 mL/min, using 25 μL of injection volume and a solvent system composed of water/acetic acid (98:2, *v/v*) (A) and methanol (B) for a total running time of 45 min. The gradient elution was as follows: 0–2 min at 5% B (initial conditions); 2–8 min linear gradient from 5% B to 25% B; 10–20 min at 40% B; 20–40 min linear gradient from 40% B to 50% B; and 0% A-100% B until the end of the run.

The UV absorption of eluates at 278 nm was recorded. The phenolic compounds were identified by comparing their retention times (*R*_t_) with those of the standards (Table S1 in Supplementary Materials). Oleacein (dialdehydic form of decarboxymethyl elenolic acid linked to hydroxytyrosol, 3,4-DHPEA-EDA) was identified tentatively according to the literature and confirmed by mass spectrometry (MS) analysis. Quantification of identified phenolic compounds was carried out by the integrated peak areas using the appropriate calibration curves with authentic standards (Table S1 in Supplementary Materials) in all cases except oleacein. Actual concentration of oleacein was calculated from the calibration curve for oleuropein, taking into account the differences in their molecular weights [12]. Phenolic compound content was expressed as mg of phenol/kg of VOO or VOO-Sea.

#### 2.3.3. MS Analysis

HPLC-MS analysis was carried out using a HPLC Agilent Technologies 1200 Series (Santa Clara, CA, USA) with binary pump, degasser, autosampler and DAD attached to Agilent Technologies 6420 Triple Quad LC/MS spectrometer equipped with electrospray ionization (ESI) source. Chromatographic separation was achieved using a C18 reversed-phase analytical column, Kinetex C18 (2.1 × 50 mm, 1.7 µm particle size, 100 Å, Phenomenex, Torrance, CA, USA). The flow rate was set at 0.5 mL/min, and the sample injection volume was 10 µL. The elution gradient consisted of mobile phase water/formic acid (99.9:0.1, *v/v*) (A) and methanol (B) for a total running time of 80 min. The gradient elution was as follows: 0–0.5 min at 90% A; 0.5–74.5 min linear gradient from 10% B to 90% B; and 90% A-10% B until the end of the run.

MS analysis was performed using a heated ESI source operating in negative ion modes (NIM) in the range *m*/*z* 50–2000 Da. The ESI needle voltage was held at ground and the capillary voltage (Vcap) set at 3500 V. Source conditions were as follows: nebuliser, 8 psi; drying gas temperature, 300 °C; drying gas flow, 8 L/min and fragmentor, 135 V. Nitrogen was used as source gas and as collision gas. The identity of oleacein, oleocanthal, oleuropein aglycone and ligstroside aglycone were confirmed by ESI-MS fragmentation profile of molecular [M-H]^−^ ions in comparison with those from the literature.

#### 2.3.4. Spectrophotometric Analysis

##### Total Phenols (TP)

TP (total phenols) concentrations in the VOO and VOO-Sea were determined spectrophotometrically at 725 nm using Folin-Ciocalteu (FC) reagent, according to Gutfinger [13]. Each sample of phenolic extract was analyzed two times. Aliquots of phenolic extract (PE) and PE-Sea were transferred to 10 mL volumetric flasks, and then water (5 mL) and FC reagent (0.25 mL) was added. The solution of saturated (20%) sodium carbonate (1.5 mL) was added to the reaction mixture after 3 min. The solution was then diluted with water to 10 mL. After 30 min, the absorbance was measured on UV-VIS spectrophotometer (Hewlett Packard 8453, Böblingen, Germany) at 725 nm against a methanol blank. Gallic acid was used as a standard for preparing the calibration curve (Table S2 in Supplementary Materials) and the amount of TP in extracts was expressed as mg gallic acid equivalent (GAE)/kg of VOO or VOO-Sea.

##### o-Diphenols

The concentration of *o*-diphenols in VOO and VOO-Sea was determined by sodium molybdate assay [14]. Each sample of phenolic extract was analyzed two times. An aliquote of 0.5 mL of extract was diluted with 5 mL methanol/water (1:1, *v/v*) to prepare a diluted extract. Then, 0.5 mL of a 5% sodium molibdate solution in methanol/water (1:1, *v/v*) was added in 2 mL of diluted extract. The content was mixed and kept in the dark (15 min) and then the absorbance was measured at 350 nm spectrophotometrically against a reagent blank. A calibration curve (Table S2 in Supplementary Materials) was obtained by measurement of standard solutions of gallic acid following the procedure described above. The concentration of *o*-diphenols in extracts was expressed as mg gallic acid equivalent (GAE)/kg of VOO or VOO-Sea.

##### Total Flavonoids (TF)

TF concentration in VOO and VOO-Sea was determined by spectrophotometric assay [15]. Each sample of phenolic extract was analyzed two times. A 1 mL aliquot of appropriately diluted extracts was added to a 10 mL volumetric flask containing 4 mL water. Then, a 5% sodium nitrite solution (0.3 mL) was added, followed by a 10% aluminum chloride solution (0.3 mL). Test tubes were incubated at ambient temperature for 5 min, and then 2 mL of 1 M sodium hydroxide was added to the mixture. The volume was immediately adjusted to 10 mL with water and thoroughly mixed. Absorbance of the mixture, pink in color, was determined at 510 nm. Catechin served as a standard for preparation of the calibration curve (Table S2 in Supplementary Materials). The concentration of TF was expressed as mg catechin equivalents (CE)/kg of VOO or VOO-Sea.

##### DPPH Test of Antioxidant Activity

The phenolic extracts, PE and PE-Sea, were analyzed according to the procedure described by Villaño et al. [16] with some modifications. Aliquots (0.1 mL) of five different concentrations of diluted PEs were added to methanolic solution of DPPH radical (2.9 mL, 7.5 × 10^−5^ M). The solution was shaken, and after 30 min kept in dark, the absorbance at 517 nm was measured. DPPH scavenging effect was calculated according to the equation: % DPPH scavenging effect = (*A*_DPPH_ − *A*_sample_)/*A*_DPPH_ × 100, with *A*_DPPH_ is the absorbance of DPPH solution, *A*_sample_ is the absorbance of the examined solution (DPPH + PE). The EC_50_ value is the concentration of TP in PE or PE-Sea (µg GAE/mL PE or PE-Sea, corresponding to 50% reduction of the initial DPPH concentration), and was obtained from the dependence of the % scavenging effect on the concentration of PE.

### 2.4. Statistical Analysis

Analysis of variance (ANOVA) test (*n* = 3), Pearson’s correlation tests (*p* < 0.01; *p* < 0.05) and principal component analysis (PCA) were performed in Python 2.7. software package using SciPy.stats library (Python Software Foundation, Beaverton, OR, USA).

## 3. Results and Discussion

### 3.1. Identification and Quantification of Phenolic Compounds

In this study the comparison of the phenolic profile of VOO prepared from immediately processed fresh olive fruits, and VOO-Sea prepared from fruits that were kept in seawater for ten days before processing, was performed. Olive fruits were collected from the same olive trees of the variety Oblica from the Ugljan isle (central part of Croatian Adriatic, near to the city of Zadar) in the 2016 season.

**Figure 1:**
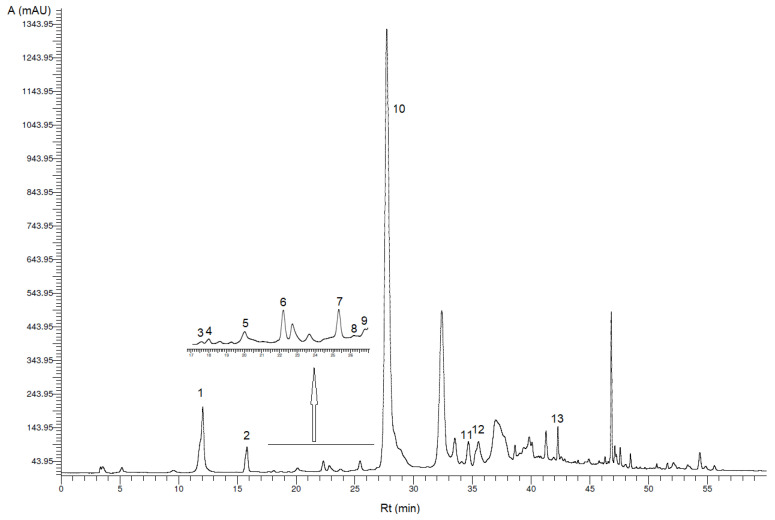
High-performance liquid chromatography (HPLC) chromatogram of PE (phenolic extract) at 278 nm. Peaks correspond to phenolic compounds in Table 1 (A—absorbance, *R_t_—*retention time).

**Figure 2:**
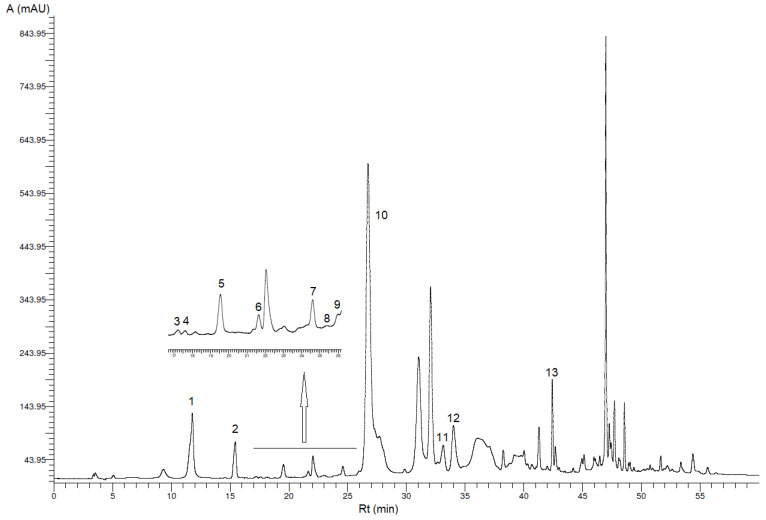
HPLC chromatogram of PE-Sea at 278 nm. Peaks correspond to phenolic compounds in Table 1 (A—absorbance, *R_t_*—retention time).

The phenolic profiles of the oils after extraction of phenolic compounds by optimized US-LLE method (Section 2.3.1) were analyzed using different methods (Section 2.3.2, Section 2.3.3, Section 2.3.4). The HPLC-DAD analysis of phenolic extracts showed several peaks corresponding to different phenolics (Figure 1 and Figure 2) determined under the chromatographic conditions described above (Section 2.3.2). Those peaks correspond to the important bioactive compounds hydroxytyrosol, tyrosol and oleacein and another 11 minor phenolic compounds. Phenolic acids like 3,4-dihydroxybenzoic acid, *o*-coumaric acid and syringic acid were below the limit of detection in VOO and VOO-Sea.

Oleacein (peak 10), the most abundant secoiridoid in all analyzed oil samples, was firstly identified by its *R*_t_ tentatively according to the literature [17]. Identity of oleacein was confirmed by MS analysis (Section 2.3.3). Analysis of fragmentation of molecules in ESI-MS spectra (Figures S1 and S2 in Supplementary Materials) also confirmed the presence of other bioactive secoiridoids (oleocanthal, oleuropein aglycone and ligstroside aglycone) in VOO and VOO-Sea but quantification was not possible (peaks were not resolved). The extracted-ion chromatograms (EICs) generated for PE (Figure S1b in Supplementary material) and PE-Sea (Figure S2b in Supplementary Materials), gave the deprotonated molecule at *m*/*z* 639, 319, 195, 183, 165, 59 which demonstrates the presence of oleacein [18]. The EIC chromatograms on Figures S1c and S2c showed *m*/*z* 607, 361, 303, 291, 285, 259, 179, 165, 139 in full-scan mode. According to the literature, these *m*/*z* 361, 291, 259, 139 peaks may correspond to ligstroside aglycone isomers (ligstroside aglycone mono-aldehyde; *p*-HPEA-EA) and *m*/*z* 607, 303, 285, 179, 165 peaks correspond to open ring decarboxylated dialdehidic form of ligstroside derivative, oleocanthal (dialdehydic form of decarboxymethyl elenolic acid linked to tyrosol; *p*-HPEA-EDA) [18]. Figures S1d and S2d show EIC chromatograms of PE and PE-Sea with fragmentation *m*/*z* 755, 377, 345, 307, 275, 139 which correspond to oleuropein aglycone (oleuropein aglycone mono-aldehyde, 3,4-DHPEA-EA) [18].

The phenolic compound concentrations (mg/kg VOO or VOO-Sea) are shown in Table 1.

The determined concentrations of hydroxytyrosol in VOO and VOO-Sea, that amount to 18.2 mg/kg and 12.9 mg/kg, respectively, are generally in the range similar to the values in some Spanish (6.9–72.7 mg/kg of oil) or Italian EVOOs (0.85–36.7 mg/kg of oil) [17,19,20], and are even higher than those previously published for Oblica variety (6.2–11.3 mg/kg of oil) [11,21,22]. Furthermore, the tyrosol concentration determined in VOO and VOO-Sea (8.10 mg/kg and 7.20 mg/kg respectively) is very similar to the values in the same literature.

The concentrations of total phenols, *o*-diphenols, total flavonoids and EC_50_ values of phenolic extracts were determined spectrophotometrically and are summarized in Table 2. The values of TP and *o*-diphenols found in VOO and VOO-Sea are frequently greater in comparison with the values of TP and *o*-diphenols in VOOs from other countries [23,24,25,26,27].

### 3.2. Effect of Storage of Olive Fruits in the Seawater on the Phenolic Profile of Olive Oil

**Figure 3:**
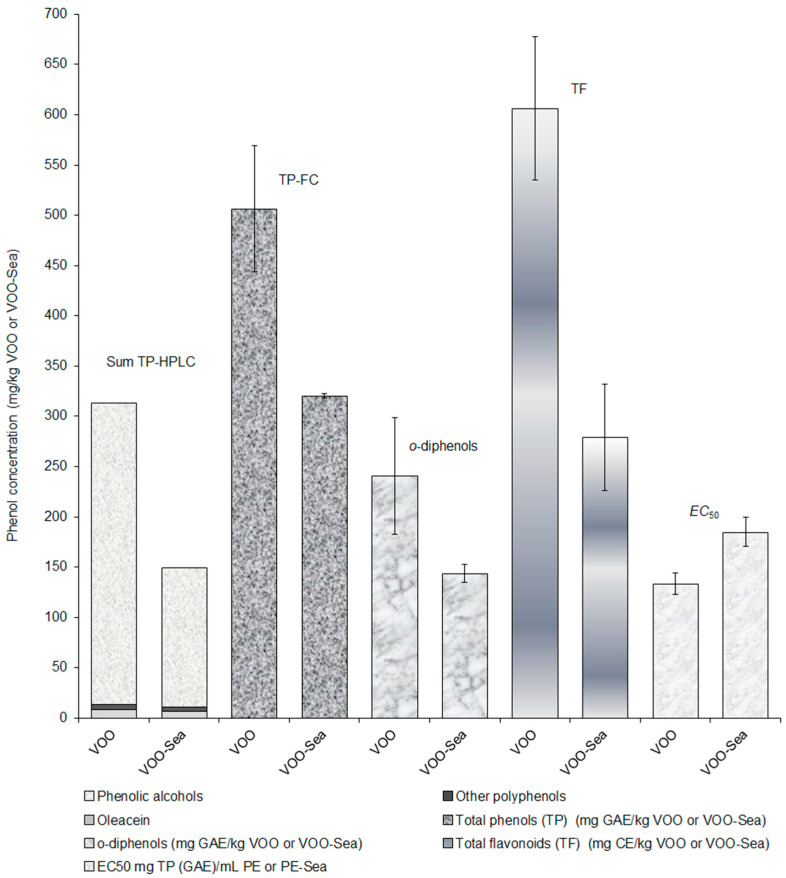
Comparison of phenolic concentrations and EC_50_ value in VOO and VOO-Sea. Sum TP-HPLC, sum of total phenols determined by HPLC-DAD analysis; TP-FC, total phenols determined by Folin-Ciocalteu analysis; TF, total flavonoids; EC_50_, concentration of total phenols in PE or PE-Sea leading to 50% reduction of the initial DPPH concentration.

The present study concentrates on the question as to whether the olives contained bioactive phenolic compounds that were eventually preserved or lost during the storage of the fruits in seawater before further processing. Inspection of Table 1 and Table 2 and Figure 3 shows that about one half of the phenolics in general were retained in the “seawater olive oil” as compared with the VOO obtained by immediate common mechanical processing of freshly picked olives.

At least with regard to the Oblica variety studied, the most abundant olive variety in Croatia, about two thirds of the total phenolic content as well as the content of the *o*-diphenols were retained in the “seawater oil”. In our opinion, there are no obvious reasons to expect a very different pattern with other varieties because we expect the epicarp of olive fruit could be of similar permeability. Similarly, the value of EC_50_, as one possible measure of antioxidant activity, is increased for VOO-Sea not more than 40 % compared to parent VOO, corresponding to not more than a 40% decrease in antioxidant activity. The sum of quantified bioactive phenolic compounds (Table 1) is reduced to half (from 334.7 mg/kg VOO to 165.6 mg/kg VOO-Sea) but it is important that hydroxytyrosol, biomedically one of the most important phenolic compounds [6] in EVOOs, and tyrosol, known also for beneficial health effects [28], are preserved to 71 and 89% of the EVOO figure, respectively. Furthermore, there is no phenolic acid or derivative that has been retained less than 58%, except in the case of vanillin where 43% of the content in EVOO remained. However, in the case of homovanillyl alcohol, *p*-coumaric acid and ferulic acid, the corresponding figures are 58, 70 and 81%, respectively. Interestingly, the “seawater oil” appears to comprise more vanillic acid than the parent EVOO (50% increase).

Oleacein, known to have antioxidant, anti-inflammatory, antiproliferative and antimicrobial activities [3,29], is found to be abundant in the examined EVOO of the Oblica variety from the isle of Ugljan; the oil is rich in this hydroxytyrosol derivative, containing 300 mg/kg, see Table 1. The “seawater oil” contains 138 mg/kg, 46% of the amount in EVOO, nevertheless a significant concentration of oleacein when compared with the literature data for the Oblica variety [22,30]. However, it is necessary to note that the average concentrations of oleacein can reach levels as high as 840 mg/kg, as was detected in some Italian EVOOs [20].

The content of pinoresinol and apigenin [4] has been modified. Moreover, the content of apigenin is 70% greater in “seawater oil”. The results correspond with research conducted by Hachicha et al. [31], whose findings suggest that the apigenin content in olive fruit increases in storage. This suggests that certain biochemical processes in olive fruit are still underway but probably at a slower pace.

Analysis of fragmentation of molecules in ESI-MS spectra confirmed the presence of bioactive secoiridoids: oleacein, oleocanthal, oleuropein aglycone and ligstroside aglycone in VOO and in “seawater olive oil”. Oleocanthal especially attracts attention, as it has been compared to ibuprofen [7] and also exerts other biological activities [3,8].

The difference in phenolic profile among VOO and VOO-Sea was moreover confirmed by statistical analysis. Correlation tests reveal different correlation profiles pertaining to a particular olive processing (Tables S3 and S4 in Supplementary Materials). PCA performed on the chemical data shows clustering of the samples on the basis of the effect of storage of olives in seawater prior to processing. Using only three PCs, 97.9% variance is retained (Figures S3 and S4 in Supplementary Materials).

Although we have determined a loss of phenolic compounds in VOO-Sea, the loss would be even greater in the case of open-air storage [31]. The enzyme activity in the fruit has probably been reduced and some of the oxidation processes have not occurred due to a lower oxygen concentration in the seawater. The presence of pathogenic bacteria and fungi in the fruit is also reduced [32].

Taken together, the comparison between the data for the two olive oils, the VOO obtained from freshly picked and immediately mechanically processed olive fruits of the Oblica variety from Ugljan isle, and the “seawater oil” obtained from the corresponding sample of olive fruits stored in seawater for 10 days prior to common processing, could suggest that despite the reduction in the content of bioactive compounds as a result of storage in seawater, its content is above the values in other varieties of olives and oils. Moreover, when olives of high quality with respect to the content of phenolics are in question, as in the case of the Oblica variety would be expected, the “seawater oils” can be competitive with regard to the biomedical usefulness taking into consideration olive varieties and oils less abundant in the phenolics. In addition, the studies on oil quality have reported no substantial differences with regard to the overall oil quality [33,34]. Insofar as the market and the habits of the customers go, “seawater oil” is still very popular.

## 4. Conclusions

The bioactive phenolic compounds in olive oil obtained from fruits stored for ten days in seawater prior to common mechanical processing into oil are partially preserved in the oil. Especially, this should be the case when the olive fruits used are of high quality with respect to the content of the important bioactive phenolics. More favorable results are expected with only several days storage, which is most frequently the case in the actual practice. Therefore, these oils, here denoted as “seawater olive oils”, should be considered as oils that are still of some biomedical relevance, although they are of somewhat less biomedical value than the parent EVOOs with high amount of the phenolics possessing favorable healthy effects.

## Figures and Tables

**Table 1:** Concentrations of phenolic compounds (mean value ± SD; *n* = 3) in VOO (virgin olive oil) and VOO-Sea (virgin seawater-stored olive oil) determined by high-performance liquid chromatography-diode array detector (HPLC-DAD) analysis.

Peak *	Phenolic Compound	VOO (mg/kg VOO ± SD)	VOO-Sea (mg/kg VOO-Sea ± SD)
1	Hydroxytyrosol	18.2 ^b^ ± 3.0	12.9 ^a^ ± 1.0
	3,4-Dihydroxybenzoic acid	n.d.	n.d.
2	Tyrosol	8.10 ^b^ ± 0.34	7.20 ^a^ ± 0.28
3	*p*-Hydroxybenzoic acid	0.10 ± 0.02 **	0.11 ± 0.00 **
4	Homovanillyl alcohol	0.38 ± 0.04 **	0.22 ± 0.01 **
5	Vanillic acid	0.64 ^a^ ± 0.20	0.97 ^b^ ± 0.03
	Syringic acid	n.d.	n.d.
6	Vanillin	0.53 ± 0.05	0.23 ± 0.01 **
7	*p*-Coumaric acid	0.43 ± 0.04 **	0.30 ± 0.04 **
8	Benzoic acid	0.22 ± 0.10 **	0.24 ± 0.07 **
9	Ferulic acid	0.27 ± 0.04 **	0.22 ± 0.02 **
10	Oleacein	300 ^b^ ± 32	138 ^a^ ± 5
	*o*-Coumaric acid	n.d.	n.d.
11	Pinoresinol	4.02 ^b^ ± 0.12	2.64 ^a^ ± 0.07
12	Cinnamic acid	0.84 ^a^ ± 0.17	0.93 ^b^ ± 0.06
13	Apigenin	0.98 ^a^ ± 0.03	1.68 ^b^ ± 0.10

* Peak in HPLC chromatogram (Figure 1 and Figure 2). ** Concentration of phenolic compound above limit of detection (LOD) but below limit of quantification (LOQ). n.d.: compound is described as not detected. Different small letters within each row denote significantly different concentrations (ANOVA test, *p* ≤ 0.05).

**Table 2:** Concentrations (mean value ± SD; *n* = 3) of phenolic compounds in VOO (virgin olive oil) and VOO-Sea (virgin seawater-stored olive oil) and EC_50_ values.

	VOO	VOO-Sea
Total phenols *	506 ^b^ ± 63	320 ^a^ ± 2
*o*-Diphenols *	241 ^b^ ± 58	144 ^a^ ± 9
Total flavonoids **	606 ^b^ ± 71	279 ^a^ ± 53
EC_50_ ***	133 ^a^ ± 11	185 ^b^ ± 15

* Concentration expressed as mg GAE/kg VOO or VOO-Sea ± SD. ** Concentration expressed as mg catechin (CE)/kg VOO or VOO-Sea ± SD. *** TP concentration (µg gallic acid equivalent (GAE)/mL PE or PE-Sea ± SD) required for the reduction of the 50 % of the initial 2,2-diphenyl-1-picrylhydrazyl (DPPH) concentration. Different small letters within each row denote significantly different concentrations (ANOVA test, *p* ≤ 0.05).

## Supplementary Materials

The following are available online at https://www.mdpi.com/2304-8158/9/10/1347/s1, Figure S1: HPLC-ESI-MS chromatograms (total ion current—TIC) of (**a**) PE (phenolic extract prepared from VOO); (**b**) 3,4-DHPEA-EDA (dialdehydic form of decarboxymethyl elenolic acid linked to hydroxytyrosol, oleacein); (**c**) *p*-HPEA-EDA (dialdehydic form of decarboxymethyl elenolic acid linked to tyrosol, oleocanthal) and *p*-HPEA-EA (ligstroside aglycone mono-aldehyde); (**d**) 3,4-DHPEA-EA (oleuropein aglycone mono-aldehyde)., Figure S2: HPLC-ESI-MS chromatograms (total ion current—TIC) of (**a**) PE-Sea (phenolic extract prepared from VOO-Sea); (**b**) 3,4-DHPEA-EDA (dialdehydic form of decarboxymethyl elenolic acid linked to hydroxytyrosol, oleacein); (**c**) *p*-HPEA-EDA (dialdehydic form of decarboxymethyl elenolic acid linked to tyrosol, oleocanthal) and *p*-HPEA-EA (ligstroside aglycone mono-aldehyde); (**d**) 3,4-DHPEA-EA (oleuropein aglycone mono-aldehyde), Figure S3: The score-plot of samples from principal component analysis (PC1 vs. PC2); VOO (▲), VOO-Sea (●), Figure S4: The score-plot of samples from principal component analysis (PC1 vs. PC3); VOO (▲), VOO-Sea (●), Table S1: HPLC-DAD analysis of phenolic compounds in VOO and VOO-Sea, Table S2: Spectrophotometric analysis of phenolic compounds in VOO and VOO-Sea, Table S3: Pearson correlation among individual phenols for VOO samples, * *p* < 0.05, ** *p* < 0.01, Table S4: Pearson’s correlation among individual phenols for VOO-Sea samples, * *p* < 0.05, ** *p* < 0.01.
