# Supplementary material for: Antique Traditional Practice: Phenolic Profile of Virgin Olive Oil Obtained from Fruits Stored in Seawater
Source: Foods. 2020 Sep 23;9(10):1347. doi: 10.3390/foods9101347 (PMC7598162; doi:10.3390/foods9101347)
Supplement: Supplementary file 1 [file foods-09-01347-s001.zip › Supplementary data Fig S1-S4 Toric et all.docx]

Article

**Antique Traditional Practice: Phenolic Profile of Virgin Olive Oil Obtained from Fruits Stored in Seawater**

Jelena Torić ^1^, Monika Barbarić ^1,^*, Stanko Uršić ^1^, Cvijeta Jakobušić Brala ^1^,
Ana Karković Marković ^1^, Maja Zebić Avdičević ^2^ and Đani Benčić ^3^

^1^ Faculty of Pharmacy and Biochemistry, University of Zagreb, A. Kovačića 1, 10000 Zagreb, Croatia; jelenatoric@gmail.com (J.T.); [dr.sursic@gmail.com](mailto:dr.sursic@gmail.com) (S.U.); cjakobus@pharma.hr (C.J.B.); [akarkovic@pharma.hr](mailto:akarkovic@pharma.hr) (A.K.M.)

^2^ Faculty of Mechanical Engineering and Naval Architecture, University of Zagreb,
Ivana Lučića 5, 10000 Zagreb, Croatia; maja.zebic@fsb.hr

^3^ Faculty of Agriculture, University of Zagreb, Svetošimunska cesta 25, 10000 Zagreb, Croatia; bencic@agr.hr

***** Correspondence: [mbarbaric@pharma.hr](mailto:mbarbaric@pharma.hr) ; Tel.: +385-01-6394-472; Fax.: +385-01-6394-400

Received: 25 August 2020; Accepted: 21 September 2020; Published: date

Supplementary data: Figure S1-S4.


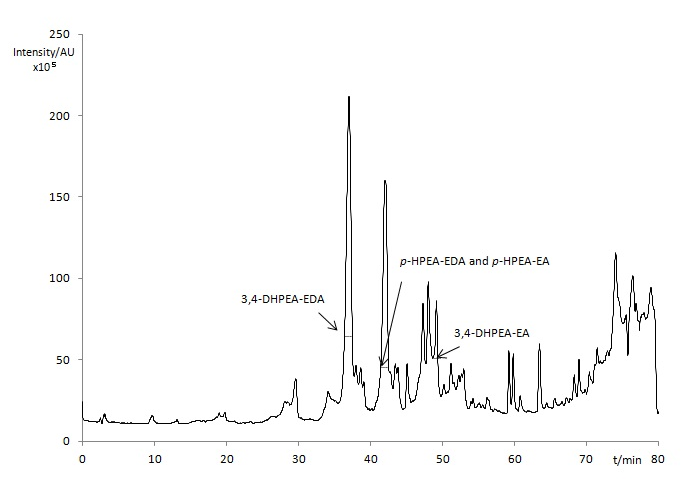


a)


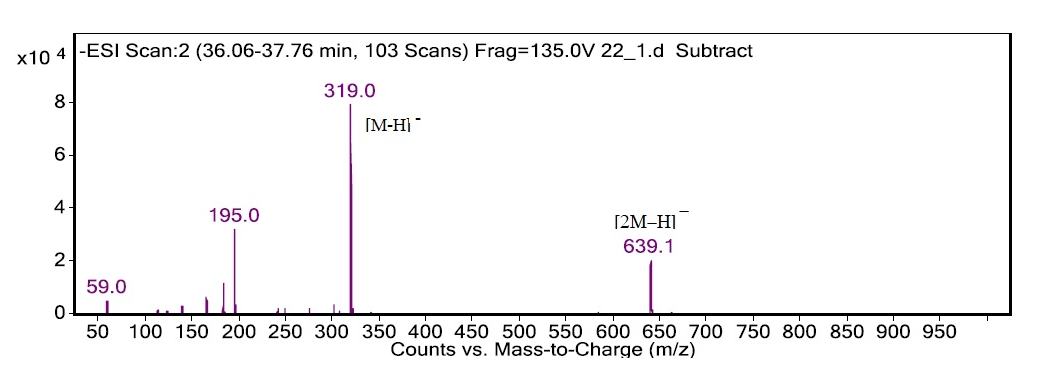


b)


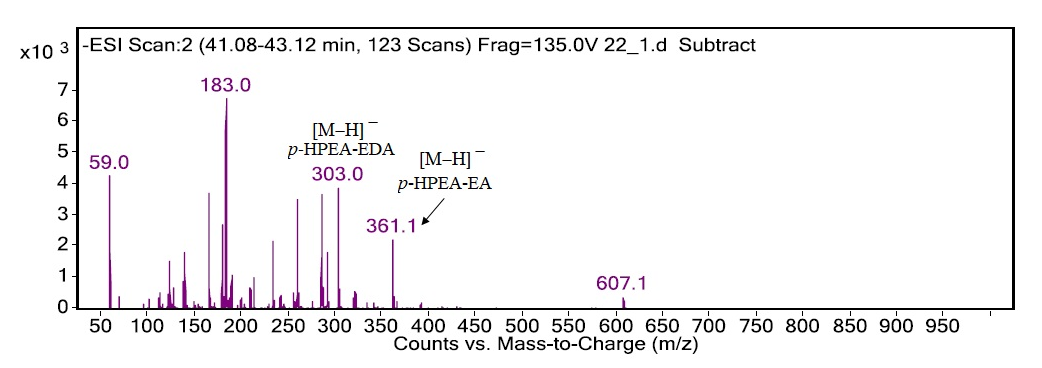


c)


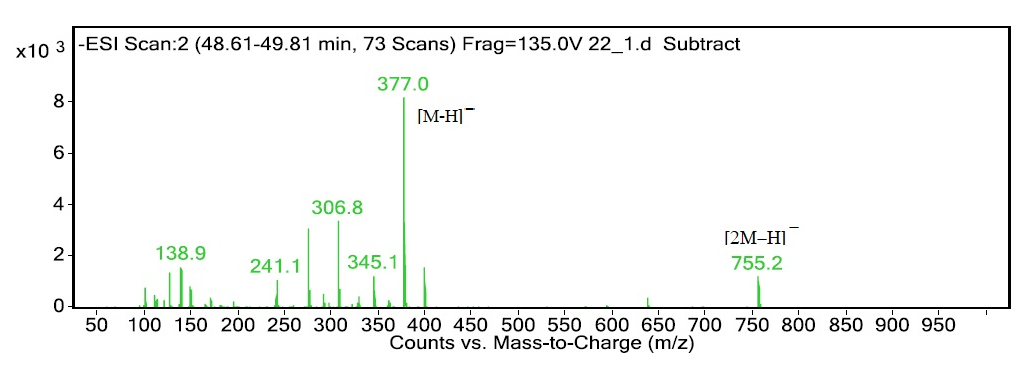


d)

**Figure S1** HPLC-ESI-MS chromatograms (total ion current—TIC) of (**a**) PE (phenolic extract prepared from VOO); (**b**) 3,4-DHPEA-EDA (dialdehydic form of decarboxymethyl elenolic acid linked to hydroxytyrosol, oleacein); (**c**) *p*-HPEA-EDA (dialdehydic form of decarboxymethyl elenolic acid linked to tyrosol, oleocanthal) and *p*-HPEA-EA (ligstroside aglycone mono-aldehyde); (**d**) 3,4-DHPEA-EA (oleuropein aglycone mono-aldehyde).


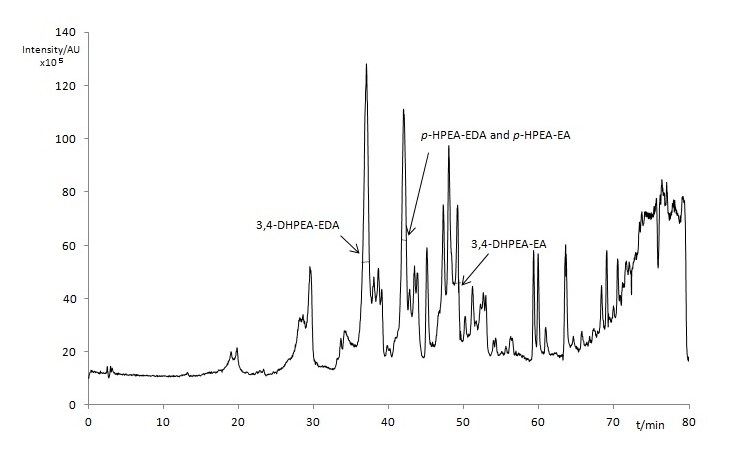


a)


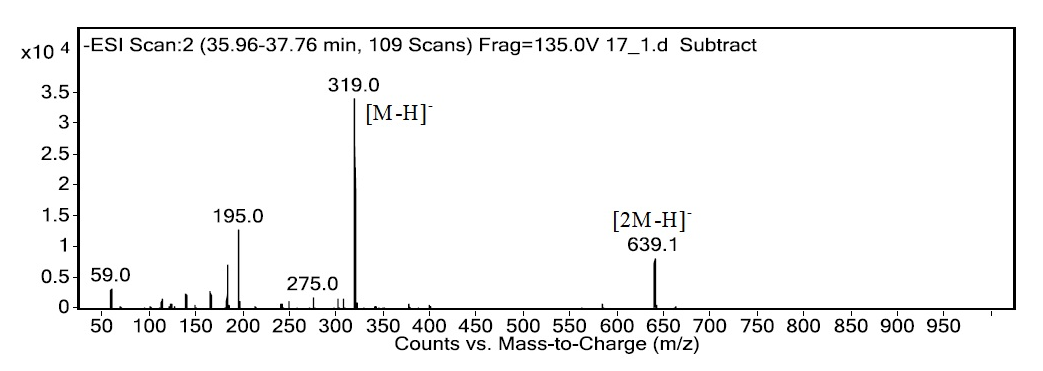


b)


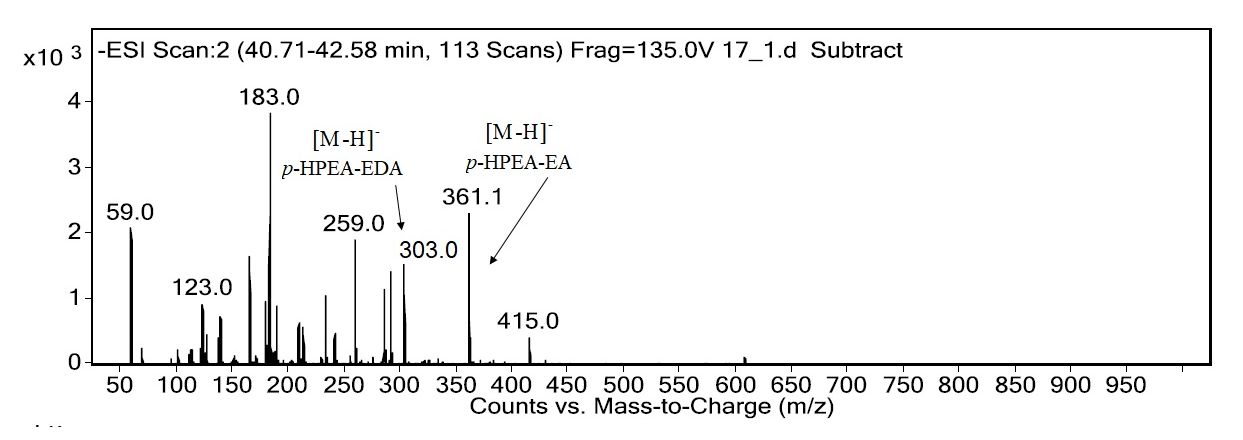


c)


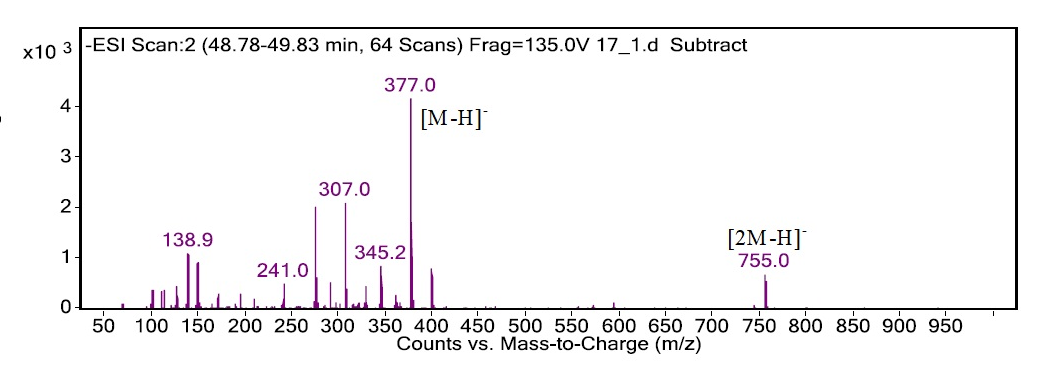


d)

**Figure S2** HPLC-ESI-MS chromatograms (total ion current—TIC) of (**a**) PE-Sea (phenolic extract prepared from VOO-Sea); (**b**) 3,4-DHPEA-EDA (dialdehydic form of decarboxymethyl elenolic acid linked to hydroxytyrosol, oleacein); (**c**) *p*-HPEA-EDA (dialdehydic form of decarboxymethyl elenolic acid linked to tyrosol, oleocanthal) and *p*-HPEA-EA (ligstroside aglycone mono-aldehyde); (**d**) 3,4-DHPEA-EA (oleuropein aglycone mono-aldehyde).


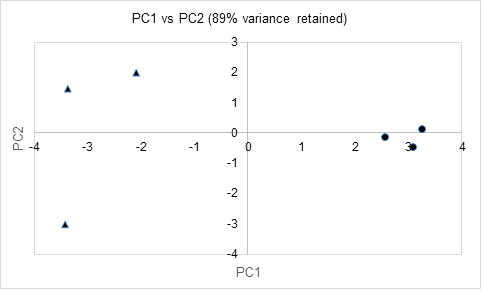


**Figure S3** The score-plot of samples from principal component analysis (PC1 vs PC2); VOO (▲), VOO-Sea (●).


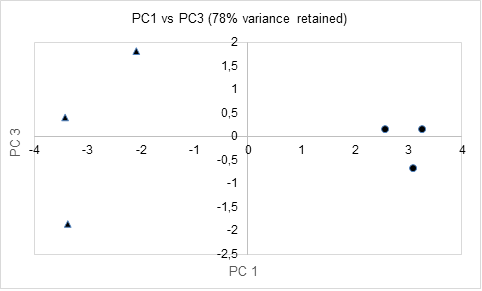


**Figure S4** The score-plot of samples from principal component analysis (PC1 vs PC3); VOO (▲), VOO-Sea (●).
